# Deciphering the genetic landscape of enhanced poly-3-hydroxybutyrate production in *Synechocystis* sp. B12

**DOI:** 10.1186/s13068-024-02548-8

**Published:** 2024-07-16

**Authors:** Anna Santin, Flavio Collura, Garima Singh, Maria Silvia Morlino, Edoardo Bizzotto, Alessandra Bellan, Ameya Pankaj Gupte, Lorenzo Favaro, Stefano Campanaro, Laura Treu, Tomas Morosinotto

**Affiliations:** 1 Department of Biology, University of Padova, 35131 Padua, Italy; 2 Waste to Bioproducts Lab, Department of Agronomy Food Natural Resources Animals and Environment, University of Padova - Agripolis, 35020 Legnaro, PD Italy; 3 Department of Microbiology, Stellenbosch University, Private Bag X1, Matieland, 7602 South Africa

**Keywords:** Cyanobacteria, Poly-3-hydroxybutyrate, Light, Starvation, Transcriptomics

## Abstract

**Background:**

Microbial biopolymers such as poly-3-hydroxybutyrate (PHB) are emerging as promising alternatives for sustainable production of biodegradable bioplastics. Their promise is heightened by the potential utilisation of photosynthetic organisms, thus exploiting sunlight and carbon dioxide as source of energy and carbon, respectively. The cyanobacterium *Synechocystis* sp. B12 is an attractive candidate for its superior ability to accumulate high amounts of PHB as well as for its high-light tolerance, which makes it extremely suitable for large-scale cultivation. Beyond its practical applications, B12 serves as an intriguing model for unravelling the molecular mechanisms behind PHB accumulation.

**Results:**

Through a multifaceted approach, integrating physiological, genomic and transcriptomic analyses, this work identified genes involved in the upregulation of chlorophyll biosynthesis and phycobilisome degradation as the possible candidates providing *Synechocystis* sp. B12 an advantage in growth under high-light conditions. Gene expression differences in pentose phosphate pathway and acetyl-CoA metabolism were instead recognised as mainly responsible for the increased *Synechocystis* sp. B12 PHB production during nitrogen starvation. In both response to strong illumination and PHB accumulation, *Synechocystis* sp. B12 showed a metabolic modulation similar but more pronounced than the reference strain, yielding in better performances.

**Conclusions:**

Our findings shed light on the molecular mechanisms of PHB biosynthesis, providing valuable insights for optimising the use of *Synechocystis* in economically viable and sustainable PHB production. In addition, this work supplies crucial knowledge about the metabolic processes involved in production and accumulation of these molecules, which can be seminal for the application to other microorganisms as well.

**Supplementary Information:**

The online version contains supplementary material available at 10.1186/s13068-024-02548-8.

## Background

The continuously increasing consumption of plastics is one of the main environmental burdens of the modern society [1]. Use of traditional petroleum-based plastics is not only unsustainable in the long term [2, 3] but also leads to severe environmental consequences, since the accumulation of plastic waste is causing devastating effects on ecosystems, wildlife and human health [4]. One alternative to common plastics are poly-hydroxyalkanoates, such as poly-3-hydroxybutyrate (PHB) which are bio-based, renewable and biodegradable polymers [5–7]. Although PHB is currently used in numerous commercial applications, presently, it is mainly produced by heterotrophic bacteria, posing a potential competition with human food-supply chain as these organisms usually require expensive organic carbon (C) sources [8–10]. One strategy to address this issue is to exploit phototrophic organisms, such as cyanobacteria, which readily fix inorganic C, with an added advantage of converting atmospheric carbon dioxide (CO_2_) into medium term C storage as plastic, mitigating its accumulation in the atmosphere [11].

The cyanobacterium *Synechocystis* is a promising candidate for PHB production owing to its fast growth rate and ease of genetic manipulation [3, 12, 13]. In fact, is a native C storing polymer, which can accumulate around 4.5% (w/w) of PHB in stationary phase, reaching up to 9.5% and 11% in nitrogen (N) and phosphorus deprivation [6, 14]. In cyanobacterial cells, PHB generate through a biosynthetic pathway starting from acetyl-CoA and involving three catalytic steps catalysed by Pha proteins [15]. The precursor acetyl-CoA is also involved in many other metabolic processes, such as glycolysis, glycogen catabolism and lipid related pathways, so that increasing C flux towards acetyl-CoA has been shown to a good strategy to improve PHB production [16].

N supply and light intensity have been identified as two major environmental factors influencing PHB production. N starvation [12] induces PHB production because, in the absence of an essential nutrient required for protein synthesis, cyanobacteria redirect the product of photosynthetic CO_2_ fixation into reserve molecules, accumulating PHB [12, 17, 18]. It serves as an electron and C sink for products of photosynthesis [19–21], enabling at the same time to maintain redox balance within the cell and avoid over-reduction of the photosynthetic electron transport chain [21, 22].

The effect of light on PHB production, on the other hand, is much less known and is suggested to be strain-specific [6, 23–28]. However, most available studies regarding PHB production used constant light intensities ranging between 30 and 150 μmol photons m^−2^ s^−1^, much lower than natural light intensity in any potential large-scale production system [28].

Despite the strong advantages of PHB production through cyanobacteria, such as low nutrient requirements, no need for organic feedstock, potential growth in industrial-scale photoreactors and low environmental impact [29, 30], there are still several constraints. Low PHB yields, high costs of downstream processing, and difficulty in achieving the best trade-off between biomass and PHB accumulation are factors that limit large-scale implementation of the process [1, 18, 31]. The cyanobacterial strain *Synechocystis* sp. B12, recently isolated from a contaminated mangrove site in Brazil, has been shown to produce high amounts of PHB, up to 241 mg L^−1^ (31% w/w), while being tolerant to high-light intensity. These features make it extremely valuable for outdoor industrial production [27] but also as model to identify the molecular bases of superior PHB accumulation.

Here, we investigate *Synechocystis* sp. B12 to identify the genetic basis of its enhanced PHB productivity. In addition to its potential interest as a platform for sustainable industrial PHB production, this information will also be seminal in increasing accumulation in other microorganisms.

## Materials and methods

### Cell culture conditions and growth experiments

*Synechocystis* sp. PCC 6803 was obtained from the Pasteur Institute (France) [25, 32], while *Synechocystis* sp. B12 was isolated from a mangrove site located in Santos (Brazil) [27]. Cultures were maintained in BG-11 medium [32], at 30 °C, agitation of 120 rpm and under 35 μmol photons m^−2^ s^−1^ continuous white light provided by LED lamps.

Cells growing in exponential phase in normal BG-11, supplemented with 6 mM NaHCO_3_, were diluted at OD_750_ of 0.3 in 60 mL Erlenmeyer flasks, and exposed to two different N conditions: normal BG-11 as control or N-free BG-11, and exposed to two different light conditions: 35 μmol photons m^−2^ s^−1^ as control low-light or 300 μmol photons m^−2^ s^−1^ as high-light. The cell growth was routinely monitored for 7 days, through OD_750_ measurements with a TECAN Spark™ Microplate Reader. Growth phase was determined by plotting the OD_750_ on a logarithmic scale.

To determine the dried biomass concentration, 5 mL were filtered on 0.45 µm pore size nitrocellulose membranes (Millipore). Nitrocellulose membranes were dried overnight in an oven at 65 °C and weighed before and after the filtration to determine the net cell dry weight (CDW).

All experiments were carried out in at least three biological replicates. The variance between the different conditions was estimated through two-way ANOVA (assuming the Gaussian distribution of data) followed by Tukey’s multiple comparison test with significance set at *p* < 0.05. Analyses were performed through the software GraphPad Prism (v.10.1).

### Pigment content determination

Chlorophyll *a* and total carotenoids were extracted from 2 mL of *Synechocystis* culture in 100% N,N′-dimethylformamide, as previously described [33]. Pigments concentrations were measured with a spectrophotometer (Cary 300 UV–Vis, Agilent) and calculated according to Ref. [34].

### PHB evaluation through Nile Red staining

Fast evaluation of PHB production by different *Synechocystis* strains in different N and light conditions was carried out using Nile Red (Merck) staining, implementing previous protocol [35]. In this case, 800 μL of cells, concentrated at OD_750_ of 0.2 in deionized water, was mixed with Nile Red to reach a final concentration of 1.25 μg mL^−1^ and incubated in the dark at 54 °C for 1 h. The fluorescence was measured using a TECAN Spark™ Microplate Reader, with excitation and emission wavelengths at 488 nm and 585 nm, respectively [35].

Fluorescence-activated cell sorting (FACS) was carried out on a BD LSRFortessa™ Cell Analyzer flow cytometer. The following parameters were observed: forward scatter, side scatter, PerCP (Peridinin chlorophyll)-Cy5.5 fluorescence and PE (Phycoerythrin) fluorescence to detect Nile Red (Additional file 1: Fig. S1).

### PHB extraction and analysis

To evaluate the PHB production, approximately, 35 mL of cyanobacterial cultures was pelleted at 3000 g for 15 min, freeze-dried and weighed. 10 mg of lyophilized cells was subjected to de-esterification using acidified (3% H_2_SO_4_) methanol in combination with chloroform in 1:1 ratio incubating for 4 h at 100 °C, and measured according to Ref. [36]. The resulting methyl esters of hydroxyalkanoates were analysed through a Thermo Finnigan Trace Gas Chromatograph, equipped with FID detector and AT-WAX column as previously reported [37]. The carrier gas was helium at the flow rate of 1.2 mL min^−1^. The split/splitless injector was used with a split ratio of 1:30 set at 250 °C, the FID temperature was 270 °C, while the oven was set at 150 °C throughout the run. Benzoic acid and poly-3-hydroxybutyric acid (Sigma-Aldrich) were adopted as internal and external standards, respectively.

### Genome sequencing, assembly and annotation

DNA extraction from *Synechocystis* sp. B12 was carried out as Ref. [27] and quantified using Nanodrop™ (ND 1000 Spectrophotometer). Illumina libraries were prepared with the Nextera DNA Flex Library Prep Kit (Illumina Inc.) and were sequenced using the Illumina Novaseq platform. Nanopore sequencing was performed using the Rapid Barcoding kit (SQK-RBK004) and a FLO-MIN106 R9 flow cell on a MinION device (Oxford Nanopore Technologies). Sequencing was performed at the next-generation sequencing facility of the Biology Department (University of Padua).

The assembly was obtained from both Nanopore and Illumina reads. Nanopore reads were assembled using Canu (v.2.2) [38]. Illumina reads were first trimmed with Trimmomatic (v.0.39) [39] and aligned using Bowtie2 (v.2.4.4) [40]. Pilon (v.1.24) [41] was used to polish the final assembly. As *Synechocystis* sp. B12 was recently isolated from the environment, CAT (v.5.2.3) [42] was used to assess the presence of contaminants in the genome. Contigs belonging to B12 were binned using Metabat2 (v.2.15) [43] and the quality of the final genome was assessed using Quast (v.4.1) [44] and CheckM2 (v.1.0.0) [45]. PLASMe (v.1.1) [46] was used to assess the presence of plasmids. Prokka [47] was used to annotate the genomes. The same gene prediction and annotation pipelines and settings were used for both the strains to identify strain-specific genes.

The single nucleotide polymorphisms (SNPs) and DNA insertions and deletions (indels) calling was done using GATK’s Haplotype Caller pipeline [48]. SnpEff (v.5.2) [49] was then used for variants annotation to predict their putative effects.

### RNA sequencing and transcriptomic analyses

RNA was extracted from *Synechocystis* sp. PCC 6803 and B12 cultures, growing with and without N in high-light conditions for 48 h, using Trireagent (Sigma-Aldrich) according to the manufacturer’s instructions. DNase I (Qiagen) treatment was applied to remove the gDNA contamination, and RNA was further purified using Direct-zol™ RNA Microprep (ZYMO Research). RNA quality was first checked by gel electrophoresis (1% agarose w/v) and then quality and quantity were verified using a Bioanalyzer™ (Agilent Technologies). Samples were treated with the QIAseq FastSelect kit (Qiagen) in order to mask ribosomal RNAs. Libraries were prepared with the Illumina Stranded mRNA Prep (Illumina inc.) and sequencing was carried out on Illumina Novaseq 6000 platform (2 × 150, paired end) at the sequencing facility of the Biology Department of (University of Padua).

The genome sequence of *Synechocystis* sp. PCC 6803 (RefSeq ID: GCF_000009725.1) and the corresponding gene annotations were downloaded from NCBI and used to perform the transcriptomic analysis. RNA-seq Illumina paired-end reads were trimmed with Trimmomatic (v.0.39) and clipped with BBDuk (v.38.86) to remove adapters. Trimmed and filtered reads were aligned to the reference genome with Bowtie2. Read count tables were obtained by extracting from the alignment files the number of reads mapping within each gene with HT-Seq [50].

Differential expression analysis was performed with DEseq2 (v.3.14) [51] by comparing PCC 6803 against B12 in both N conditions. Benjamini–Hochberg correction for *p* values was applied to each gene [52]. Enrichment analysis was performed using iDEP (v.0.96) [53].

## Results

### Effect of light and nitrogen supply on cyanobacterial growth

*Synechocystis* sp. B12 physiological properties were compared to the reference strain *Synechocystis* sp. PCC 6803. Growth and biomass productivity were assessed during a growth experiment under low- or high-light (35 or 300 μmol photons m^−2^ s^−1^, respectively), in N-replete or N-free medium.

**Fig. 1:**
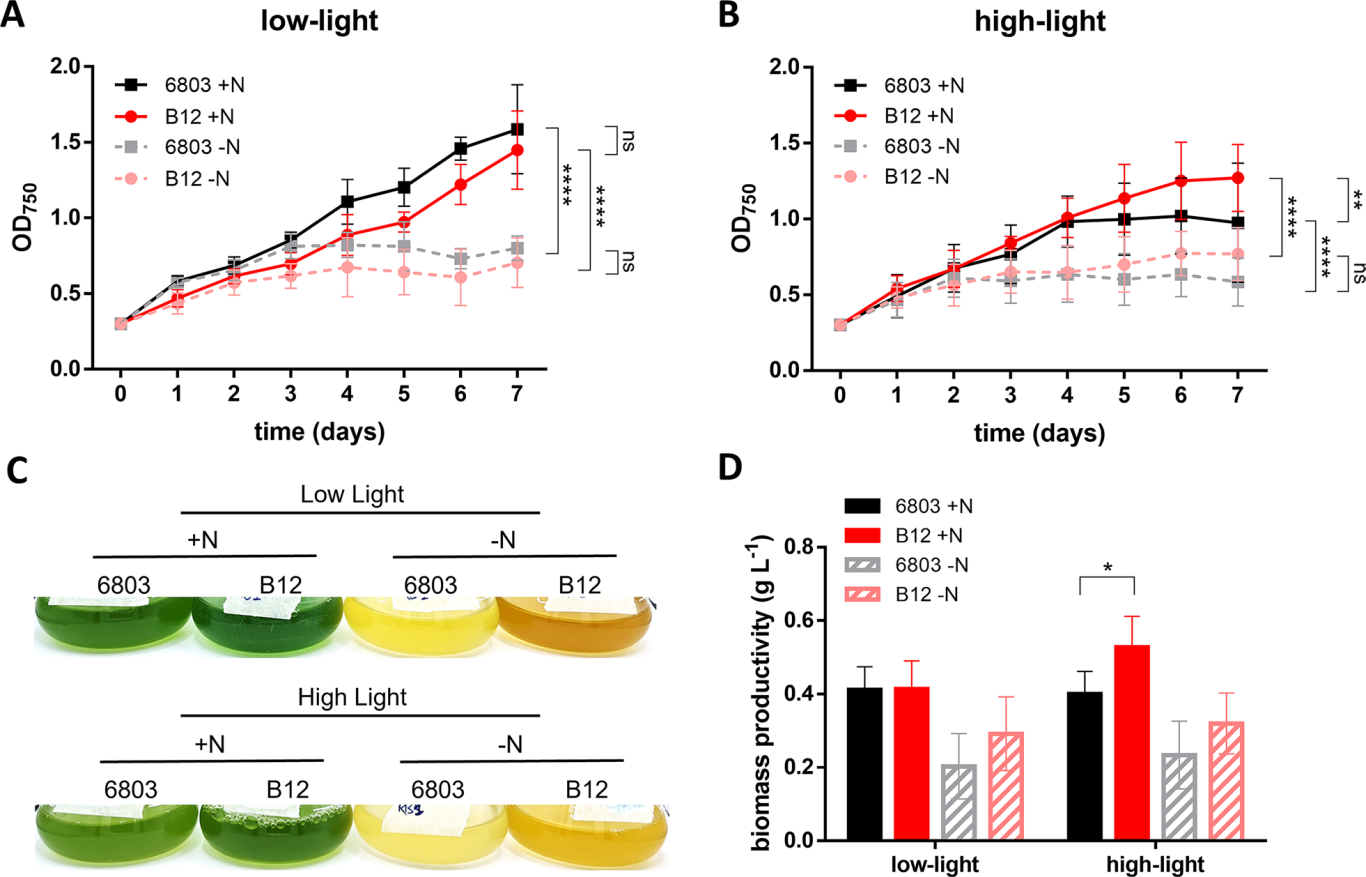
Growth and biomass of the *Synechocystis* sp. strains PCC 6803 and B12. **A**, **B** Graphs show the optical density measured at 750 nm (OD_750_) of *Synechocystis* sp. strains growing in BG-11 medium N-replete or N-free, in low-light conditions at 35 μmol photons m^−2^ s^−1^ (**A**) and in high-light conditions at 300 μmol photons m^−2^ s^−1^ (**B**). Data indicate the average (± SD) of at least three biological replicates. (**C**) Photos of PCC6803 and B12 cultures grown for 7 days in BG-11 medium N-replete or N-free, in low- and high-light conditions. (**D**) Whole biomass production, represented as g L^−1^, including both cell and PHB biomass of PCC 6803 and B12, grown in BG-11 medium N-replete or N-free, in low- and high-light conditions. Data indicate the average (± SD) of at least three biological replicates. * indicates *p* < 0.05, **p < 0.01, ***p < 0.001 and ****p < 0.0001 (for complete statistical output, see Table S1)

In low-light conditions, as expected, there was a growth difference depending on N in the medium. Cells of both strains growing in N-free medium reached the stationary phase after three days, while cells growing in N-replete medium were still in late exponential phase after 7 days, reaching double concentrations with respect to N-free growing cells (Fig. 1A, C). This trend was confirmed by biomass quantifications (Fig. 1D).

In cultures exposed to high-light, growth in N-free medium was similar to that in low light (Fig. 1B–D) in the two strains, suggesting the nutrient depletion is the parameter with the highest impact on growth. In N-replete medium, a detrimental effect of high-light was noticeable in *Synechocystis* sp. PCC 6803, which reached the stationary phase after 4 days, as opposed to 6 days for strain B12 (Fig. 1B, C). Overall, *Synechocystis* sp. B12 presented an increased growth compared to PCC 6803 in N-replete medium and high-light condition, in particular in the second part of the experiment (Fig. 1B, D). This was confirmed by *Synechocystis* sp. B12 higher biomass productivity under strong illumination, approximately 25% higher than PCC 6803 in the same condition (Fig. 1D).

**Fig. 2:**
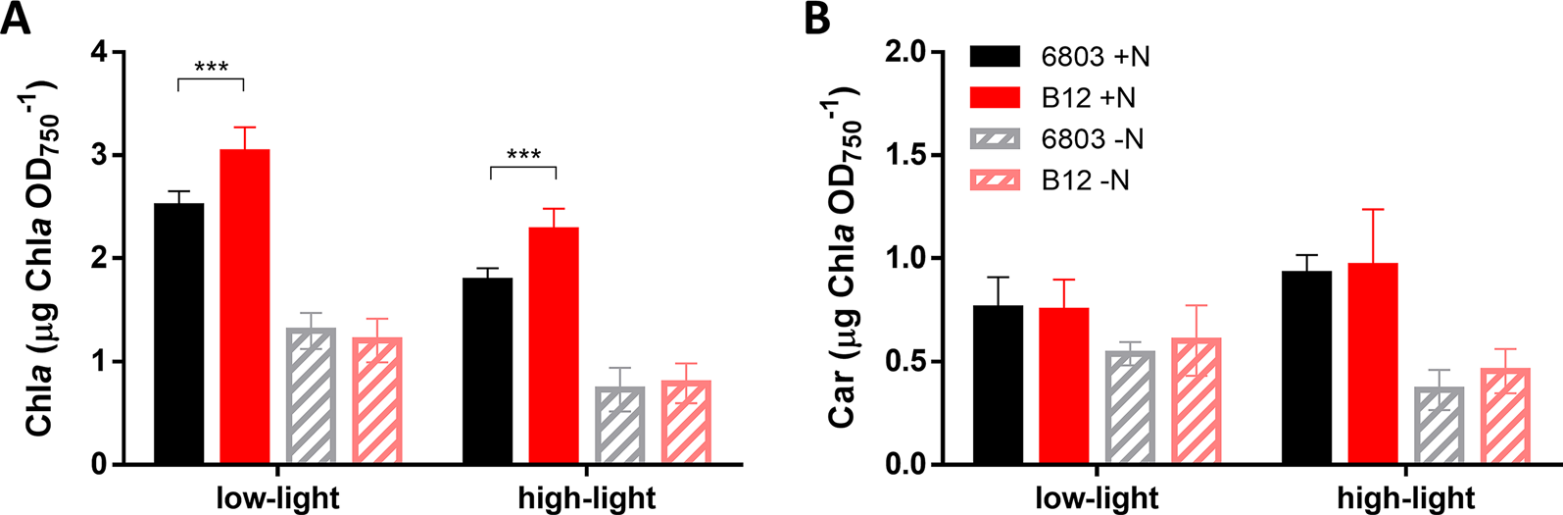
Pigment content of *Synechocystis* sp. PCC 6803 and B12, normalised on the OD_750_. **A** Chlorophyll *a* and **B** carotenoid content of *Synechocystis* sp. strains growing in BG-11 medium N-replete or N-free, in low-light conditions at 35 μmol photons m^−2^ s^−1^ and in high-light conditions at 300 μmol photons m^−2^ s.^−1^, after 7 days from the beginning of the experiment. Data indicate the average (± SD) of at least three biological replicates. *** indicates *p* < 0.001 (for complete pigment analysis, see Additional file 1: Fig. S2)

Since culture colour was strongly different based on experimental conditions (Fig. 1C), pigments content was also investigated. Under N starvation, both in low- and in high-light conditions, both strains showed a much lower pigment content than with full media, with limited impact from illumination intensity (Fig. 2A, B and Additional file 1: Fig. S2). N-replete conditions showed instead a larger difference between strains, with a higher chlorophyll *a* content in *Synechocystis* sp. B12 compared to the reference strain in both light conditions (Fig. 2A and Additional file 1: Fig. S2).

### PHB production in response to different conditions

**Fig. 3:**
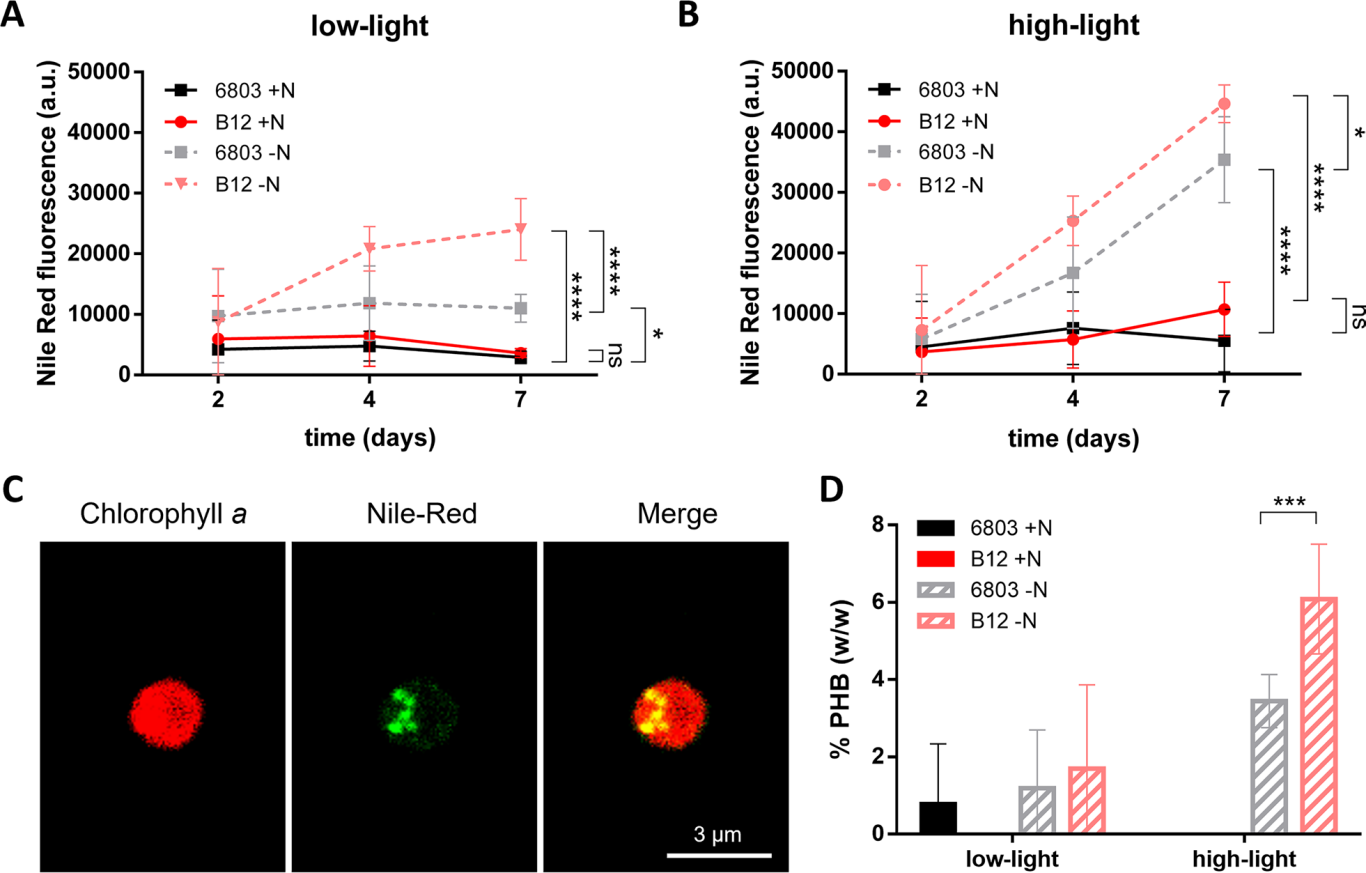
PHB production of *Synechocystis* sp. PCC 6803 and B12 cells. **A**, **B** Nile Red fluorescence normalised on OD_750_, used as proxy for PHB production, after 2, 4 and 7 days from the beginning of the experiment. *Synechocystis* sp. strains were grown in BG-11 medium N-replete or N-free, in low-light conditions at 35 μmol photons m^−2^ s^−1^ (**A**) and in high-light conditions at 300 μmol photons m^−2^ s.^−1^ (**B**). **C** Confocal images of a *Synechocystis* sp. B12 cell grown in N starvation for 7 days, stained with Nile Red highlighting PHB granules. Red indicates chlorophyll autofluorescence, green for Nile Red-stained PHB granules. Scale bar: 3 µm. **D** Quantification of PHB as percentage of cell dry weight. Data indicate the average (± SD) of at least three biological replicates. * indicates *p* < 0.05, ** *p* < 0.01, *** *p* < 0.001 and **** *p* < 0.0001 (for complete statistical output, see Table S1)

PHB accumulation was periodically assessed by Nile Red staining, to have a fast indication of intracellular polymer content during the experiment. When growing in N-replete medium, whether in low- or high-light conditions, PHB production was negligible in both strains independent of light conditions (Fig. 3A, B), suggesting light alone is not sufficient to stimulate PHB accumulation. In contrast, when grown under N starvation, both strains produced PHB, with *Synechocystis* sp. B12 showing higher amount of intracellular PHB in comparison to PCC 6803, in both light conditions (Fig. 3A). These PHB were intracellularly accumulated as granules, which can be observed through microscopy (Fig. 3C). High-light in combination with N starvation stimulated an even higher PHB production by *Synechocystis* sp. B12, 1.5 times higher than in low-light (Fig. 3A, B).

Nile Red observations were confirmed by chromatographic PHB quantification after 7 days of growth (Fig. 3D), with both strains showing higher PHB production in N-starved condition in high l-ght, reaching 3.4% of CDW in *Synechocystis* sp. PCC 6803 and 6.1% in *Synechocystis* sp. B12 strain (Fig. 3D).

### Genome assembly and variant analysis

To identify the genetic bases for the differential PHB accumulation capacity and high-light growth in *Synechocystis* sp. B12 strain, its genome was sequenced. The assembled genome comprised four scaffolds, of which one corresponded to the chromosome, 3,660,711 bp long, and the others to three plasmids: pSYSG of 66,146 bp, pSYSM of 124,281 bp and pSYSA of 112,855 bp. The total GC content of the assembly was 47.33%. Genome completeness was 95.7% with a negligible amount of contamination (0.6%). The genome size was comparable to the one of the reference strain PCC 6803 (3.57 Mb), while the number of coding sequences (CDS) and proteins were slightly higher than the reference strain: 4402 CDS vs 3340 and 4352 proteins vs 3599 (Additional file 2).

Annotated genomes of *Synechocystis* sp. B12 and PCC 6803 were compared to identify genomic differences (Additional files 2 and 3). Reciprocal BLAST revealed the presence of strain-specific genes, most of which hypothetical (Additional file 2). Variant analyses revealed the presence of 677 non-synonymous polymorphisms between strains, out of which 150 were missense. Interestingly, no effective non-synonymous SNPs were found in genes directly involved in PHB pathway (Additional file 2). However, a deletion in B12 strain was identified in the gene encoding for the phosphate-specific transport system integral membrane protein A (*pstA*), strictly associated with PHB accumulation [54]. In fact, a point mutation in this gene was shown to be linked to an increase in PHB production in *Synechocystis* sp. PCC 6714 strain [54]. Besides higher PHB production, the mutant strain exhibited increased biomass productivity, but unaltered regulation of PHB biosynthesis-related genes, suggesting that the increased PHB production was due to genes indirectly involved in PHB synthesis, likely mediated by the downregulation of *pstA*.

### Gene expression remodelling in *Synechocystis* sp. strains

As reported in Fig. 3, N availability was found to be the main assessed factor impacting PHB accumulation in cyanobacteria. To identify the differences that make *Synechocystis* sp. B12 more efficient in this process, the mRNAs of *Synechocystis* sp. PCC 6803 and B12 strains growing in high-light, in both N-replete and N-free conditions, were sequenced.

**Fig. 4:**
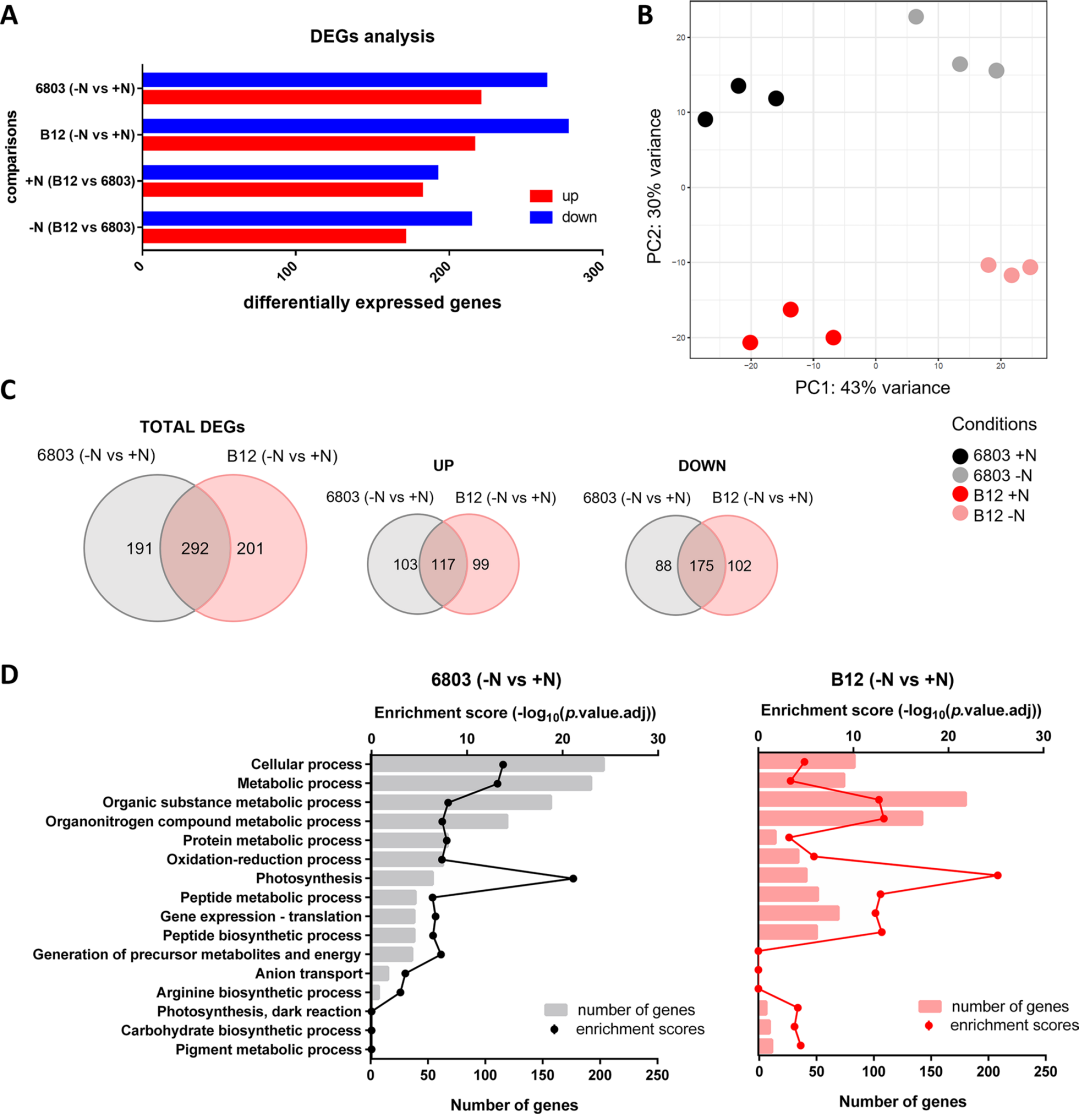
Overview of the transcriptome of *Synechocystis* sp. PCC 6803 and B12. **A** Numbers of differentially expressed genes (DEGs) for all comparisons. In the “− N vs +N” comparison “+N” indicates N-replete condition and was set as reference, while “− N” indicates N starvation. **B** Principal component analysis (PCA) shows the effect of N starvation for component 1 and changes related to the different strain for component 2. **C** Venn diagrams and clustering analysis of RNA-seq results, combining single comparisons to find strain-specific genes regulated in N starvation. **D** Functional enrichment analysis of DEGs in *Synechocystis* sp. PCC 6803 (left) and B12 (right), where the normal N condition was considered as the reference and the N starvation was the test. Y-axis indicates affected pathways, upper X-axis represents the enrichment score, represented by dots, while lower X-axis displays a number of DEGs, shown as bars

In total, 1372 genes showed statistically significant differential expression in at least one comparison. To focus on more relevant expression changes, only the genes with absolute Log_2_ Fold-Changes (LFC) ≥ 1 were considered to perform different comparisons (Fig. 4A). The principal component analysis (PCA) shows that samples of both strains are separated along the first principal component (43% of variance) according to N starvation, and along the second one (30% of variance) according to the strain (Fig. 4B).

While there was a significant similarity between the two strains, with 292 total shared differentially regulated genes (DEGs), a substantial number of genes were identified to be specifically up- (*n* = 99) and downregulated (*n* = 102) in *Synechocystis* sp. B12, suggesting a differentiated metabolic remodelling (Fig. 4C, D).

### Transcriptional variances underscoring *Synechocystis* sp. B12 advantage in high-light

**Fig. 5:**
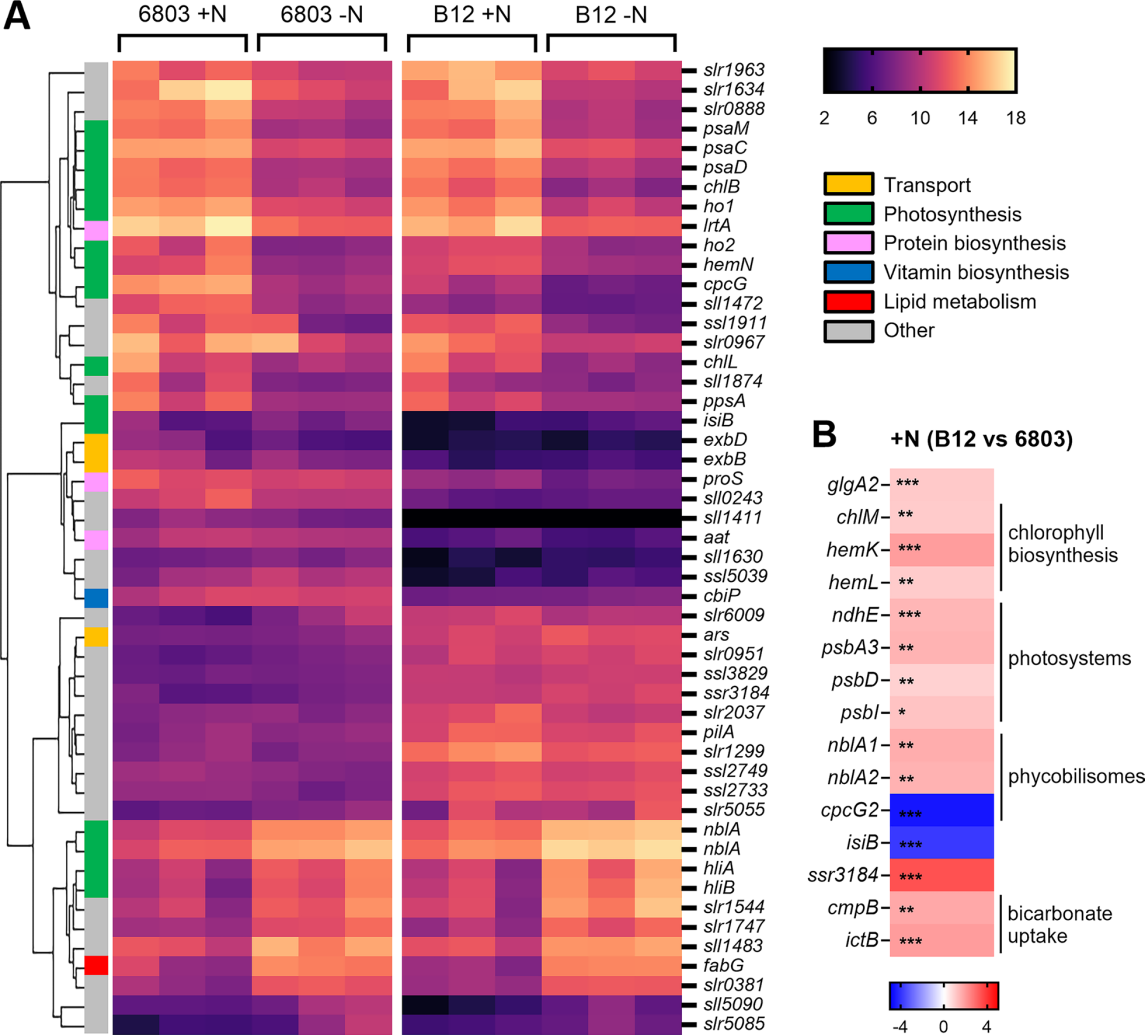
Gene expression differences between *Synechocystis* sp. PCC 6803 and B12 in high-light. **A** Heatmap of the top 50 differentially expressed genes in *Synechocystis* transcriptome. The intensity of the orange/purple colours represents the normalised expression level. The rows have been sorted according to the gene clustering tree following Pearson correlation. The left-hand bar is colour-coded according to the functions of the genes. **B** Heatmaps representing gene expression differences between strains in normal N conditions for a set of significantly (*p* < 0.05) variable (LFC > 1) genes. In this comparison, B12 expression values are reported using PCC 6803 as reference. Red shadows indicate upregulated genes while blue is used for downregulated genes, with the scale bar showing LFCs. * indicates *p* < 0.05, ** *p* < 0.01 and *** *p* < 0.001

The genes undergoing a more evident transcriptional regulation in both strains were related to photosynthesis (Figs. 4D, 5A). In particular, *hliA* and *hliB* genes were upregulated in both N-starved strains (Fig. 5A): they encode for high-light-inducible proteins protecting the photosynthetic apparatus of cyanobacteria from light stress, and known to be induced also by nutrient stress [55, 56].

Interestingly, genes which undergo higher upregulation in *Synechocystis* sp. B12 than PCC 6803 are not annotated (Fig. 5A), so their function has not been assessed on inferred yet. Thus, gene expression differences on the comparison + N (B12 vs 6803) were systematically analysed to identify variations between strains in high-light and N-replete conditions (Fig. 5B). Most of these differences referred to photosynthesis, with the upregulation of genes related to Photosystem II (PSII) (*psb* genes) and chlorophyll biosynthesis in *Synechocystis* sp. B12 (Fig. 5B), consistently with the higher measured chlorophyll content (Fig. 2B). In addition, bicarbonate transporters (*cmpB* and *ictB* genes) were significantly upregulated in B12 strain, as well as *nblA* genes involved in phycobilisome degradation (Fig. 5B). Phycobilisomes are the major light-harvesting apparatus in cyanobacteria, that decrease in number when exposed to high-light stress to reduce the amount of harvested light [57]. The upregulation of *nblA* genes in *Synechocystis* sp. B12, coupled with the downregulation of *cpcG2* encoding for a phycobilisome core linker polypeptide (Fig. 5B), suggested that the strain B12 could be more capable of modulating its light-harvesting efficiency.

### Transcriptional differences induced by N starvation

#### Photosynthesis-related genes

**Fig. 6:**
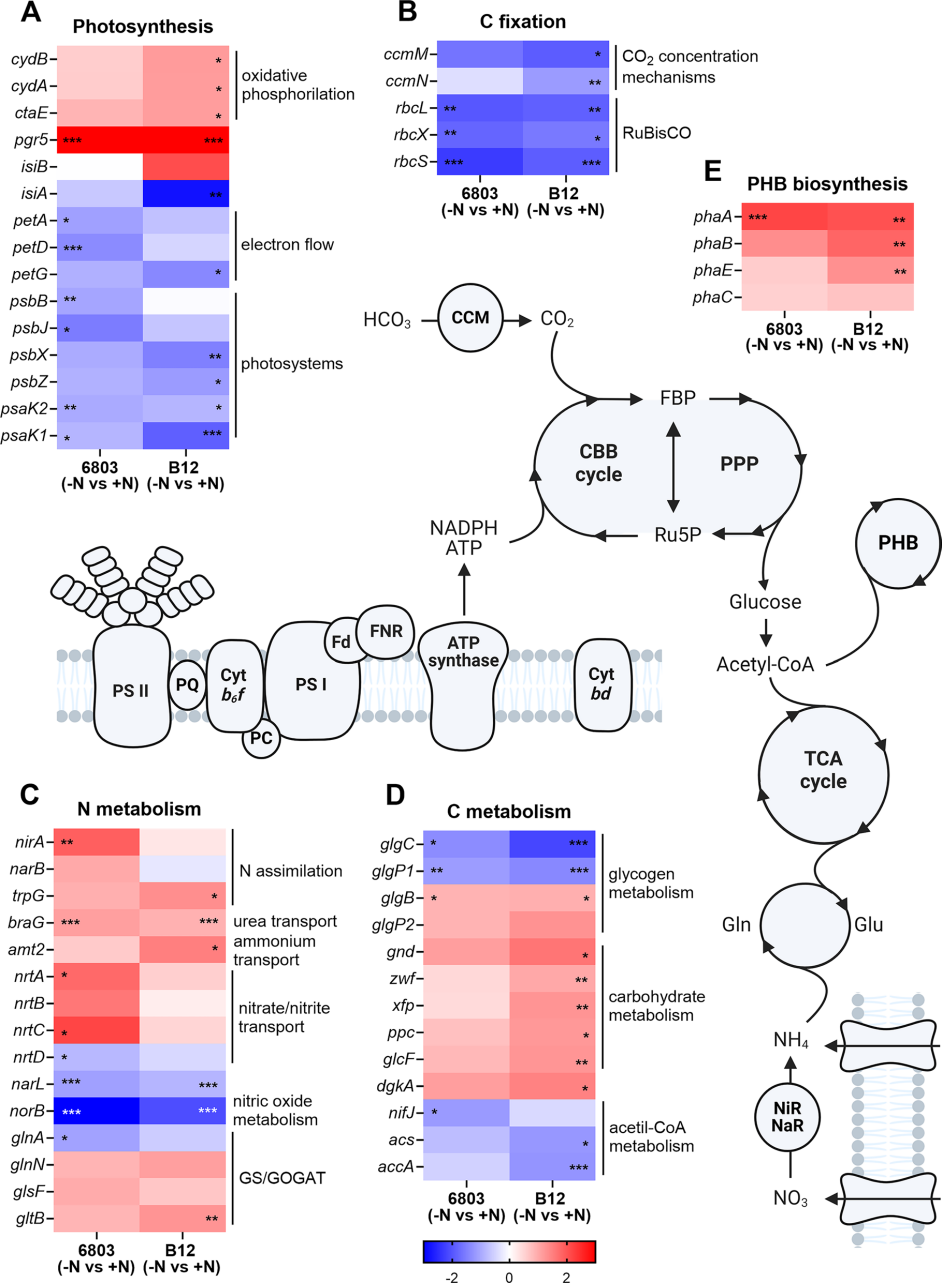
Schematic *Synechocystis* sp. metabolisms, with associated heatmaps. Heatmaps represent expression values for a set of significantly (*p* < 0.05) variable (LFC > 1) genes in the comparison B12 (−N vs +N) on the right, and the corresponding expression values in the comparison 6803 (−N vs +N) on the left. Differentially expressed genes are grouped according to the pathway: **A** photosynthesis, **B** carbon fixation, **C** nitrogen metabolism, **D** carbon metabolism and **E** PHB biosynthesis. Red shadows indicate upregulated genes while blue is used for downregulated genes, with the scale bar showing LFCs. * indicates *p* < 0.05, ** *p* < 0.01 and *** *p* < 0.001. PSII, photosystem II; PQ, plastoquinone; Cyt *b*_*6*_*f*, cytochrome *b*_*6*_*f*; PSI, photosystem I; Fd, ferredoxin; FNR, Fd-NADP^+^ oxidoreductase; Cyt *bd*, cytochrome *bd*; CCM, carbon concentrating mechanism; CBB cycle, Calvin–Benson–Bassham cycle; PPP, pentose phosphate pathway; FBP, fructose 1,6-bis-phosphate; Ru5P, ribulose 5-phosphate; PHB, poly-hydroxy-butyrate biosynthesis; TCA cycle, tricarboxylic acid cycle; Gln, glutamine; Glu, glutamate; NiR/NaR, nitrite/nitrate reductases; NH_4_, ammonium; NO_3_, nitrate

Differences in gene expression between strains in N starvation were analysed by comparing *Synechocystis* sp. 6803 (−N vs +N) with B12 (−N vs +N). It is well known that photosynthesis represents one of the most regulated pathways in N starvation, this is confirmed by the downregulation of most genes involved in allophycocyanin (*apc*), phycocyanin structure and biosynthesis (*cpc*, *hem* and *ho*), PSI (*psa*) and ATP synthase (*atp*), similar in both strains (Fig. 5 and Additional file 3: File S2). However, PSII-related genes showed different downregulation patterns: *psbB*, encoding for the chlorophyll-binding CP47 protein [58], was significantly downregulated only in N-starved *Synechocystis* sp. PCC 6803 (Fig. 6A). Another component of the core complex of PSII, *psbJ* was significantly downregulated only in PCC 6803, but *psbX* and *psbZ* were downregulated only in *Synechocystis* sp. B12 (Fig. 6A).

*PetA*, *petD* and *petG* are component of the cytochrome *b*_*6*_*f* complex, mediating electron transfer between PSII and PSI [60, 61]: while the first two were significantly downregulated in PCC 6803, *petG* was downregulated in *Synechocystis* sp. B12 (Fig. 6A and Additional file 3). As the cytochrome *b*_*6*_*f* complex is retained to be the limiting step of photosynthetic electron transport [62], a reduced downregulation in B12 could explain the higher photosynthetic capacity. Cytochrome *b*_*6*_*f* also participates in cyclic electron transfer [63], as well as *pgr5*, strongly upregulated in both strain in N starvation, and probably involved in protecting photosynthetic apparatus under stress, similarly to N-starved eukaryotic algae [64] (Fig. 6A and Additional file 3).

Other two strongly regulated genes were *isiA* and *isiB*, encoding for chlorophyll antenna protein and flavodoxin, respectively, and responsible for *Synechocystis* protection from photooxidative stress [65, 66]. *IsiB* was upregulated while *isiA* was significantly downregulated in N-starved *Synechocystis* sp. B12 (Fig. 6A). Analysing genes involved in C fixation and metabolism, CO_2_ concentration mechanisms (*ccm*) were significantly downregulated in *Synechocystis* sp. B12 in N starvation, more than in PCC 6803, even if RuBisCO-related genes (*rbc*) were similarly downregulated in both strains (Fig. 6B).

#### Regulation of genes involved in N assimilation

Previous functional enrichment analyses showed that, beside photosynthesis, metabolic processes associated with N compounds were also strongly affected (Fig. 5). Nitrate (*narB*) and nitrite reductases (*nirA*) were upregulated in N-starved PCC 6803 (Fig. 6C), similarly to nitrate/nitrite transporters *nrtA*, *nrtB* and *nrtC* (Fig. 6C and Additional file 1: Fig. S3A), indicating that PCC 6803 in N starvation could increase its potential in nitrate uptake and assimilation. On the other hand, N-starved *Synechocystis* sp. B12 specifically upregulated ammonium transporter *amt2* (Fig. 6C and Additional file 1: Fig. S3A), as well as putative amino acid transporters *slr1735*, *sll1270* and *slr0360* (Additional file 1: Fig. S3A), inferred by sequence homology, which are often used by organisms as an alternative route to take up N from the external environment [67, 68]. This could be the result of both nitrate absence in the medium and the affected biosynthesis of amino acids, which cells try to overcome by increasing the activity of the glutamine synthetase–glutamine:2-oxoglutarate aminotransferase (GS-GOGAT) cycle, through the upregulation of *glnN*, *glsF* and *gltB* (Fig. 6C). This could represent an advantage in conditions of complete N depletion like here, where in absence of other N sources, N is mainly recycled by degrading proteins. Thus, increasing the uptake of alternative N sources from the external environment could partially avoid intracellular protein degradation.

Regarding other transport systems, phosphate transport showed an upregulation in both N-starved strains [69] (Additional file 1: Fig. S3A), while sulphate as well as many other ion transporters (*kdpB*, *brtC* and *mntB*) were upregulated specifically in N-starved PCC 6803 (Additional file 1: Fig. S3A), suggesting different strategies in managing ion homeostasis.

#### Regulation of genes involved in PHB biosynthesis and acetyl-CoA metabolism

*Synechocystis* sp. B12 showed a significant upregulation of pentose phosphate pathway (PPP) in N starvation, with the induction of *gnd*, *zwf* and *xfp* genes (Fig. 6D), which couple glucose turnover to the production of pentoses and NADPH as reducing equivalents [70]. In fact, PPP generates NADPH and glyceraldehyde-3-phosphate starting from 6-phosphogluconate. These products have been shown to flow towards acetyl-CoA, and then to PHB biosynthesis in *Cupriavidus necator*, thanks to the induction of the same *gnd* and *zwf* genes [71, 72]. On the opposite, *acs* and *accA* encoding for an acetyl-CoA synthetase and an acetyl-CoA carboxylase were strongly downregulated in N-starved *Synechocystis* sp. B12 (Fig. 6D), as well as genes involved in fatty acid and phospholipid metabolism (Additional file 3). This suggested that the B12 strain could remodel its metabolism to redirect the C present in glucose to pentoses instead of lipids, ultimately favouring PHB biosynthesis.

This was confirmed by expression levels of genes involved in PHB production (Fig. 6E). The PHB biosynthetic pathway has three basic steps, which in *Synechocystis* are catalysed by *phaA*, *phaB* and *phaE*/*phaC* genes [73]. While *phaA* was upregulated in both strains in N starvation, *phaB* and *phaE* were upregulated only in *Synechocystis* sp. B12 (Fig. 6E), consistent with a stronger increase of PHB production (Fig. 3) [74].

#### Regulation of *Synechocystis* sp. B12 strain-specific genes

Genome exploration of *Synechocystis* sp. PCC 6803 and B12 revealed 26 unique genes in B12 strain (Additional file 3), that were not detected in the reference strain. Among them, only two showed a significantly different gene expression (*p* < 0.05): GKLPKBBB_04043, encoding for a putative RNA polymerase-associated protein RapA and GKLPKBBB_04033, encoding for a putative endoribonuclease MazF, both induced in N starvation (Additional file 3). *MazF* is a well-characterised gene in the bacterium *Escherichia coli*, where it regulates cell growth in response to stresses [75, 76] and its activity could contribute in B12 growth modulation in nutrients starvation.

## Discussion

The research of new bio-based and biodegradable materials, specifically those having similar characteristics of traditional plastics, is attracting more attention in recent years in the quest of developing a more sustainable production system. In this context, *Synechocystis* sp. B12 is an interesting organism because of its high-light resistance and its ability to accumulate large amounts of PHB [27]. In this work, we describe how this strain can boost the PHB production in specific conditions, by analysing *Synechocystis* sp. B12 physiological, genomic and transcriptomic features. Beyond its direct applicability as production strain, the comparison of *Synechocystis* sp. B12 with a less performing reference strain is instructive to identify molecular mechanisms responsible for high PHB accumulation and high-light tolerance, seminal for biotechnological improvements and also potentially applicable in other microorganisms.

In the assembled genome, number of coding sequences and proteins were slightly higher in *Synechocystis* sp. B12 than the reference strain, which could be explained by the use of a more updated database for the annotation of B12 strain. Despite this, a few strain-specific genes were, however, identified (Additional file 2). Ribonuclease *vapC2*, for instance, was present only in PCC 6803: this gene has been shown to be negatively associated with growth in *E. coli*, as its overexpression resulted in inhibition of bacterial growth [77]. This could suggest that the absence of *vapC* in *Synechocystis* sp. B12 may partially be responsible of its faster growth rate (Fig. 1).

### *Synechocystis* sp. B12 has a more efficient response to high-light

Unlike most cyanobacteria, *Synechocystis* sp. B12 demonstrated a remarkable resistance to intense light exposure, with an enhanced growth and chlorophyll content in B12 strain, coupled with a PHB production higher than the reference strain (Figs. 1, 2, 3). The high biomass productivity in high-light conditions makes this strain an advantageous candidate for scaling up cultivation in outdoor industrial photobioreactors [78].

Transcriptional analysis deepened how the reorganisation of the photosynthetic apparatus could explain these differences. *Synechocystis* has been already shown to downregulate phycobilisome-related genes as a strategy to decrease light-harvesting ability [79–82]. In addition, PSII and PSI are usually downregulated in high-light [80–82], with only exception of *psbA* genes, well known to have a high turnover because of light induced damage [83]. B12 carries out these responses more efficiently than the reference strain. Also, bicarbonate transporters, generally upregulated in high-light [80] were even more expressed in *Synechocystis* sp. B12, probably providing to cells more C supply that is highly helpful in reducing the perceived high-light stress by improving the capacity of using light energy for metabolisms [84].

In summary, both *Synechocystis* sp. B12 and the reference strain PCC 6803 exhibited similar responses to high-light conditions. However, B12 strain’s superior growth in high-light could be attributed to a faster or more efficient response, rather than a different mechanism.

### N starvation affects *Synechocystis* sp. B12 transcriptional regulation of photosynthesis

Transcriptomic analysis shed light on the different metabolic remodelling triggered by N starvation between strains. As expected, N depletion strongly affected photosynthesis, with most genes significantly downregulated in both strains. In fact, during starvation, light-harvesting complexes are degraded and photosynthetic activity declines together with the breakdown of thylakoid membranes [20, 85, 86]. This downregulation was strongly evident for genes encoding structural subunits of phycobilisomes, cytochrome *b*_*6*_*f* and PSI. This response was confirmed from previous transcriptomic analyses previously performed in N starvation conditions [87, 88], who cultured *Synechocystis* sp. PCC 6803 at illumination intensity of 70 μmol photons m^−2^ s^−1^, but is in contrast with others [89], who used lower light intensity, at 45 μmol photons m^−2^ s^−1^, indicating the marked influence of light on N starvation response.

The expression of *isiAB* operon was strongly regulated in *Synechocystis* sp. B12 (Fig. 6). Its transcription is known to be induced by iron starvation [65] but also other stress conditions [90, 91]. Over the years, extensive research was done on these genes, showing that the *isiA* product probably protects PSI from excessive excitation, but their function has not been clearly determined [66, 92–94]. The strong regulation of *isiA* and *isiB* in *Synechocystis* sp. B12 N-starved cells suggests that photoprotection mechanisms act differently in this strain as compared to PCC 6803. An interpretation for *isiA* downregulation of B12 strain, in high-light N starvation, could be linked to the overall decrease of PSI in these cells. In fact, high levels of *isiA* transcription are induced by strong illumination in N-replete cells, which possess a huge IsiA pool not always coupled to PSI [66, 90, 95]. In N starvation, with the downregulation of genes encoding PSI subunits and the consequent decrease in their content, there should be less need of IsiA antennas: this could result in *isiA* downregulation, with all IsiA produced coupled to PSI and a beneficial redirection of N and C resources to other pathways, similarly to the coupled decrease of PSII and phycobilisomes [90]. Results suggests that this regulation should be more flexible and efficient in *Synechocystis* sp. B12, and probably is one of the reasons of its success in N starvation when acclimated to high-light conditions.

### *Synechocystis* sp. B12 applies different N assimilation strategies to overcome N limitation

N starvation strongly affects N metabolism, from the uptake of N compounds to the assimilation into amino acids [88, 89]. We observed a general downregulation of genes involved in translation and protein synthesis in N deprivation, as previously reported [88, 89].

Strong changes in transcript levels were observed for genes involved in N uptake and metabolism, similarly to previous studies [89]. In fact, *Synechocystis* cells often induce the high-affinity nitrate uptake system under N limited conditions [88]. This was true for PCC6803 but not for the B12 strain, which instead upregulated ammonium and amino acid transporters. This metabolic remodelling suggests a different physiological strategy in the two strains in response to N starvation, with *Synechocystis* sp. B12 investing no longer on residual nitrate uptake but rather on other N sources.

This strategy could be associated with different environmental conditions and adaptation to different N sources in their cultivation environment. Being *Synechocystis* sp. B12 an isolated strain from a polluted area, it could have been exposed to ammonium-rich conditions. In fact, polluted areas are often abundant in N compounds, such as ammonia, nitrous oxides and nitrogen oxide, which can lead to a range of environmental issues like eutrophication [96]. Irrespective from the evolutionary reason, this property could be valuable enabling the B12 strain a better ability to recycle amino acids and other N products instead of degrading intracellular protein during N starvation [67, 68]. In addition, the direct uptake of amino acid has already been shown to be upregulated in different microorganisms to sustain the GS-GOGAT cycle and to maintain the amino acids’ metabolism in N starvation [20].

### *Synechocystis* sp. B12 upregulates PHB biosynthesis in N starvation

In the context of large-scale cultivation, productivity is a multifaceted outcome determined both by the biomass and the content of the desired product [27], in this case PHB. This study revealed a significant increase in PHB accumulation in *Synechocystis* sp. B12 under high-light and N depletion. In particular, N starvation is known to induce chlorosis and protein degradation [21, 26, 97], because of the need to remobilize the N present in biomolecules such as pigments and amino acids [88] and to store energy in forms of reduced carbon molecules not containing N, such as glycogen and PHB. *Synechocystis* sp. B12 showed a superior capacity to PHB accumulation, making it an interesting strain to identify the molecular determinants for this ability.

The two *Synechocystis* strains showed a different regulation of pathways involved in C metabolism, and in particular in PHB biosynthesis, under nutrient starvation. After C incorporation into glucose through the Calvin–Benson–Bassham (CBB) cycle, intermediates of carbohydrate metabolism increase at the beginning of N starvation and gradually decrease during prolonged starvation [20]. *Synechocystis* can catabolize glucose via two parallel operating pathways: the glycolysis and the PPP. The latter is strongly linked with PHB production, providing C skeletons and reducing equivalents needed for PHB biosynthetic pathway [21]. The strong induction of PPP-related genes in N-starved *Synechocystis* sp. B12, together with the upregulation of PHB biosynthetic pathway, suggests that this strain could be more efficient in directing C from carbohydrates towards acetyl-CoA and then to PHB. This is also supported by the downregulation of acetyl-CoA downstream reactions in B12 N-starved cells, suggesting the remodelling of its metabolism to reduce lipid biosynthesis and to favour C incorporation into pentoses. Together, these data indicate that B12 should be more flexible in adjusting its metabolism by favouring PHB accumulation, through the metabolization of pentoses, similar to what previously seen in bacteria and microalgae [98, 99].

## Conclusions

Our study revealed a higher production of PHB in *Synechocystis* sp. B12 strain than the reference strain, particularly under high-light and N-starved conditions. Even if few differences were observed at genome level, many interesting alterations in the expression of crucial genes for photosynthesis, N and C metabolism were described. This calls for an ongoing adaptation of the B12 strain, influenced by environmental factors in its natural habitat. These conditions likely triggered regulatory responses, shaping gene expression without necessarily altering the genome, as part of an adaptive process to specific environmental conditions.

These findings not only deepen our understanding of responses favouring PHB production in B12, but also indicate potentially broad applications extendable to other organisms. Our research sets the groundwork for future practical implementations, emphasising how these discoveries can make a big impact on eco-friendly development of novel bioplastics.

### Supplementary Information

Supplementary Material 1.

Supplementary Material 2.

Supplementary Material 3.

## Data Availability

The raw sequencing data and assembly used in this study are available under BioProject PRJNA1065842. Further data supporting the conclusions of this article will be made available by the authors upon reasonable request.

## Abbreviations

PHB: Poly-3-hydroxybutyrate
C: Carbon
CO_2_: Carbon dioxide
N: Nitrogen
OD: Optical density
CDW: Cell dry weight
SNP: Single nucleotide polymorphism
CDS: Coding sequence
LFC: Log2 fold-changes
PCA: Principal component analysis
DEG: Differentially regulated gene
PSII: Photosystem II
PQ: Plastoquinone
Cyt: Cytochrome
PSI: Photosystem I
Fd: Ferredoxin
FNR: Fd-NADP^+^ oxidoreductase
CCM: Carbon-concentrating mechanism
CBB: Calvin–Benson–Bassham cycle
PPP: Pentose phosphate pathway
TCA cycle: Tricarboxylic acid cycle
Gln: Glutamine
Glu: Glutamate
NiR/NaR: Nitrite/nitrate reductases
NH_4_: Ammonium
NO_3_: Nitrate
GS-GOGAT: Glutamine synthetase–glutamine:2-oxoglutarate aminotransferase
