# Supplementary material for: Deciphering the genetic landscape of enhanced poly-3-hydroxybutyrate production in Synechocystis sp. B12
Source: Biotechnol Biofuels Bioprod. 2024 Jul 16;17:101. doi: 10.1186/s13068-024-02548-8 (PMC11253406; doi:10.1186/s13068-024-02548-8)
Supplement: Supplementary file 1 — Supplementary Material 1. [file 13068_2024_2548_MOESM1_ESM.pdf]

SUPPLEMENTARY MATERIAL

Deciphering the genetic landscape of enhanced poly-3-hydroxybutyrate production in *Synechocystis* sp. B12

Anna Santin, Flavio Collura, Garima Singh, Maria Silvia Morlino, Edoardo Bizzotto, Alessandra Bellan, Ameya Pankaj Gupte, Lorenzo Favaro, Stefano Campanaro, Laura Treu, Tomas Morosinotto

File S1. Genome assembly statistics comparing *Synechocystis* sp. B12 with the reference strain PCC 6803. (A) Summary statistics related to B12 genome assembly. (B) B12 genome annotations. (C-D) List of unique genes retrieved in B12 (C) and PCC 6803 (D) genomes.

File S2. Complete overview of the entire transcriptome of *Synechocystis* sp. PCC and B12 strains. Transcriptome performed during nitrogen starvation (-N vs +N) in high-light. A) information includes IDs, annotations, categories, Log_2 Fold-Changes (LFCs) and *p* values adjusted for all genes regulated in the comparisons 6803 (-N vs +N), B12 (-N vs +N) and +N (B12 vs 6803). FLCs above ±1 and *p* values adjusted < 0.05 are considered significant. B) List of B12 unique genes, with corresponding LFCs and *p* values adjusted in the comparison B12 (-N vs +N).

Figure S1. Output of Nile Red staining through flow cytometry. Example of dot plots (A and D) and histograms (B, C, E and F) from a sample of *Synechocystis* sp. cells grown in conditions of N starvation (high-light) which enable PHB accumulation (D-F), and from a sample of the same B12 strain grown in conditions of N repletion (high-light) as a negative control for PHB accumulation (A-C). Chlorophyll *a* was estimated by the fluorescence (expressed in RFU, Relative Fluorescence Units) registered through the channel PerCP-Cy5-5, while the cellular PHB content estimated by the fluorescence of Nile red registered by the channel PE. Cyanobacterial cells growing in N limited medium show lower chlorophyll *a* fluorescence (E) and higher Nile Red fluorescence (F) than N-repleted cells (B-C), as observed in the peak shift indicated with red dotted lines and red arrows.

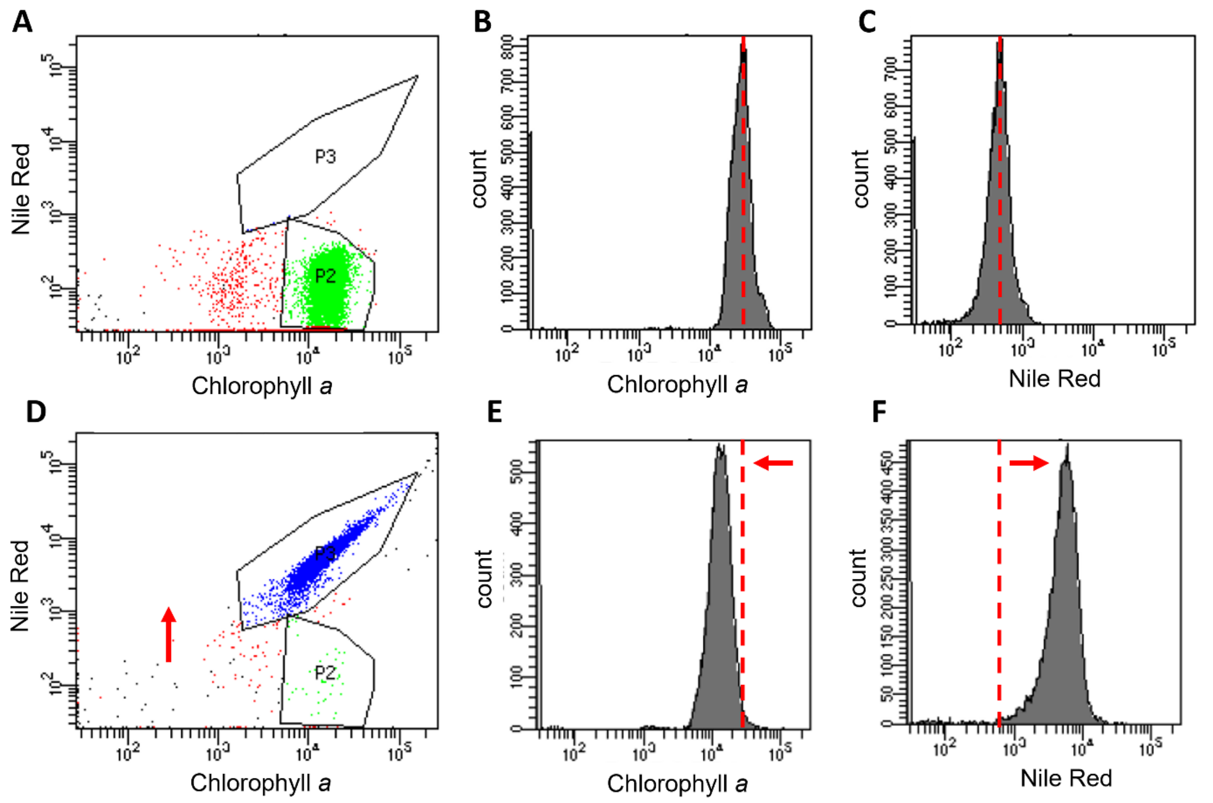

Figure S2. Pigment content of *Synechocystis* sp. PCC 6803 and B12. Pigment content measured after 2, 4 and 7 days from the beginning of the growth experiments, normalized on the OD_{750}. (A-B) Chlorophyll *a* content of *Synechocystis* sp. strains growing in BG-11 medium N-repleted or N-free, in low-light conditions at 35 µmol photons m^{-2} s^{-1} (A) and in high-light conditions at 300 µmol photons m^{-2} s^{-1} (B). (C-D) Carotenoid content of *Synechocystis* sp. strains growing in BG-11 medium N-repleted or N-free, in low-light conditions (C) and in high-light conditions (D). (E-F) Ratio between carotenoid and chlorophyll *a* content of *Synechocystis* sp. strains growing in BG-11 medium N-repleted or N-free, in low-light conditions (E) and in high-light conditions (F). Data indicate the average (±SD) of at least three biological replicates. For complete statistical output, see S1 Table.

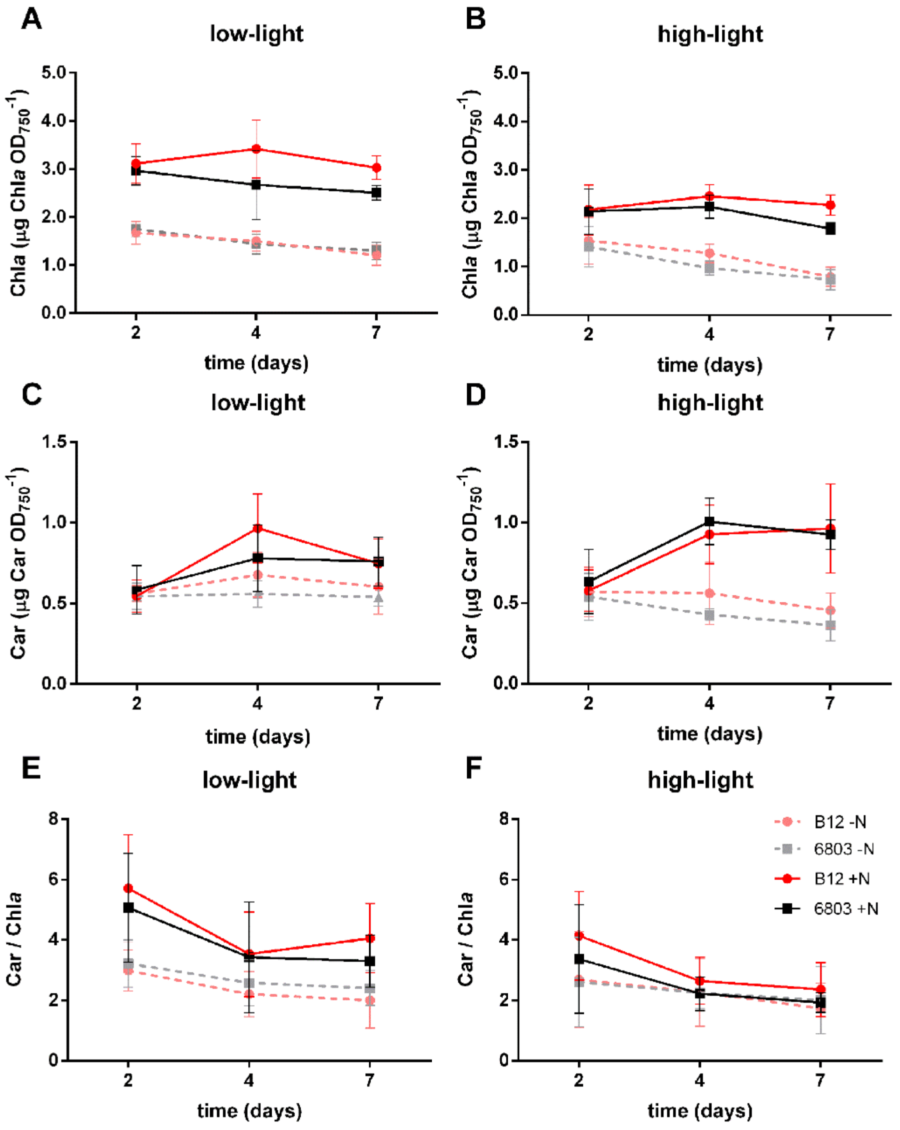

Figure S3. Heatmap of expression values for genes involved in transport and regulation. Heatmap of expression values for a set of significantly ($p < 0.05$) variable (LFC > 1) genes in the comparison B12 (-N vs +N) on the right, and the corresponding expression values in the comparison 6803 (-N vs +N) on the left. Differentially expressed genes are grouped per classes: (A) transport of different molecules and (B) genes involved in regulatory processes and responses. Red shadows indicate upregulated genes while blue is used for downregulated genes. * indicates $p <$ 0.05, ** $p < 0.01$, *** $p < 0.001$ and **** $p < 0.0001$.

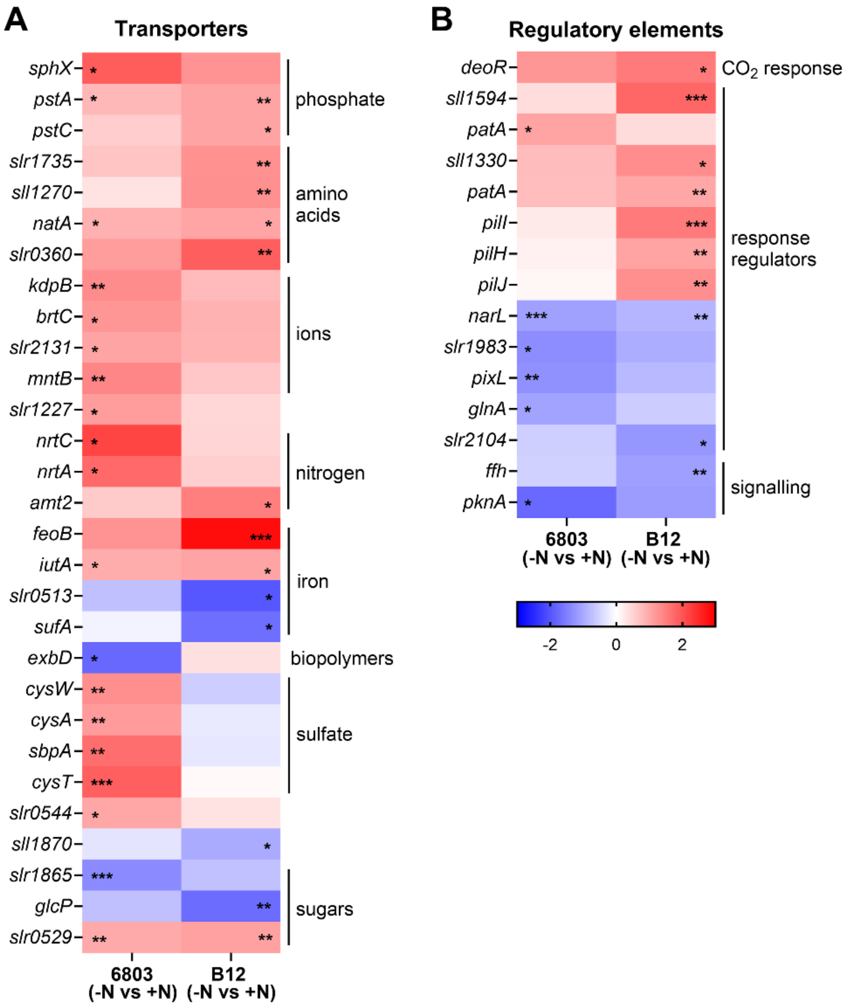

Table S1. Complete statistic output of the two-way ANOVA statistical analyses. Statistical analyses performed on OD_{750}, Nile-Red fluorescence, biomass and PHB productions, and pigment content measurments during the experiment described.

2-way ANOVA on **OD_{750} at Low-Light**. Time: 53.67 % variance, $p < 0.0001$; Strain: 18.68 % variance, $p < 0.0001$; Interaction: 17.54 % variance, $p < 0.0001$.

	Time (days)							
	0	1	2	3	4	5	6	7
6803 +N vs. B12 +N	ns	ns	ns	*	**	***	***	ns
6803 +N vs. 6803 -N	ns	ns	ns	ns	****	****	****	****
6803 +N vs. B12 -N	ns	ns	ns	***	****	****	****	****
B12 +N vs. 6803 -N	ns	ns	ns	ns	ns	*	****	****
B12 +N vs. B12 -N	ns	ns	ns	ns	**	****	****	****
6803 -N vs. B12 -N	ns	ns	ns	**	ns	*	ns	ns

2-way ANOVA on **OD_{750} at High-Light**. Time: 44.9 % variance, $p < 0.0001$; Strain: 17.98 % variance, $p < 0.0001$; Interaction: 10.47 % variance, $p < 0.0001$.

	Time (days)							
	0	1	2	3	4	5	6	7
6803 +N vs. B12 +N	ns	ns	ns	ns	ns	ns	*	**
6803 +N vs. 6803 -N	ns	ns	ns	ns	***	****	****	****
6803 +N vs. B12 -N	ns	ns	ns	ns	***	**	*	ns
B12 +N vs. 6803 -N	ns	ns	ns	*	****	****	****	****
B12 +N vs. B12 -N	ns	ns	ns	ns	***	****	****	****
6803 -N vs. B12 -N	ns	ns	ns	ns	ns	ns	ns	ns

2-way ANOVA on **Nile-Red fluorescence at Low-Light**. Time: 4.32 % variance, $p = 0.0090$; Strain: 45.81 % variance, $p < 0.0001$; Interaction: 13.388 % variance, $p = 0.0002$. 2-way ANOVA on **Nile-Red fluorescence at High-Light**. Time: 28.36 % variance, $p < 0.0001$; Strain: 34.33 % variance, $p < 0.0001$; Interaction: 19.00 % variance, $p < 0.0001$. d=days.

	Low-Light			High-Light		
	2d	4d	7d	2d	4d	7d
6803 +N vs. B12 +N	ns	ns	ns	ns	ns	ns
6803 +N vs. 6803 -N	ns	*	*	ns	ns	****
6803 +N vs. B12 -N	ns	****	****	ns	****	****
B12 +N vs. 6803 -N	ns	ns	*	ns	*	****
B12 +N vs. B12 -N	ns	****	****	ns	****	****
6803 -N vs. B12 -N	ns	**	****	ns	ns	*

2-way ANOVA on **Biomass Productivity**. Light: 2.51 % variance, $p = 0.0552$; Strain: 57.53 % variance, $p < 0.0001$; Interaction: 3.27 % variance, $p = 0.1848$. 2-way ANOVA on **PHB production**. Time: 10.11 % variance, $p < 0.0001$; Strain: 46.14 % variance, $p < 0.0001$; Interaction: 19.26 % variance, $p < 0.0001$. LL=low-light, HL=high-light.

	Biomass production		PHB production	
	LL	HL	LL	HL

6803 +N vs. B12 +N	ns	ns	ns	ns
6803 +N vs. 6803 -N	****	****	ns	****
6803 +N vs. B12 -N	*	*	ns	****
B12 +N vs. 6803 -N	****	****	ns	****
B12 +N vs. B12 -N	*	*	*	****
6803 -N vs. B12 -N	ns	ns	ns	***

2-way ANOVA on **Chlorophyll *a* at Low-Light**. Time: 3.28 % variance, p = 0.0002; Strain: 80.24 % variance, p < 0.0001; Interaction: 1.54 % variance, p = 0.2094. 2-way ANOVA on **Chlorophyll *a* at High-Light**. Time: 7.59 % variance, p < 0.0001; Strain: 66.72 % variance, p < 0.0001; Interaction: 5.35 % variance, p = 0.0027. d=days.

	Low-Light			High-Light		
	2d	4d	7d	2d	4d	7d
6803 +N vs. B12 +N	ns	***	*	ns	ns	*
6803 +N vs. 6803 -N	****	****	****	****	****	****
6803 +N vs. B12 -N	****	****	****	**	****	****
B12 +N vs. 6803 -N	****	****	****	****	****	****
B12 +N vs. B12 -N	****	****	****	***	****	****
6803 -N vs. B12 -N	ns	ns	ns	ns	ns	ns

2-way ANOVA on **Carotenoids at Low-Light**. Time: 17.45 % variance, p < 0.0001; Strain: 19.23 % variance, p < 0.0001; Interaction: 12.11 % variance, p = 0.0057. 2-way ANOVA on **Carotenoids at High-Light**. Time: 5.55 % variance, p = 0.0010; Strain: 45.95 % variance, p < 0.0001; Interaction: 17.33 % variance, p < 0.0001. d=days.

	Low-Light			High-Light		
	2d	4d	7d	2d	4d	7d
6803 +N vs. B12 +N	ns	*	ns	ns	ns	ns
6803 +N vs. 6803 -N	ns	*	*	ns	****	****
6803 +N vs. B12 -N	ns	ns	ns	ns	****	****
B12 +N vs. 6803 -N	ns	****	*	ns	****	****
B12 +N vs. B12 -N	ns	***	ns	ns	****	****
6803 -N vs. B12 -N	ns	ns	ns	ns	ns	ns

2-way ANOVA on **ratio between Chlorophyll *a* and Carotenoids at Low-Light**. Time: 15.78 % variance, p < 0.0001; Strain: 29.05 % variance, p < 0.0001; Interaction: 3.01 % variance, p = 0.5660. 2-way ANOVA on **ratio between Chlorophyll *a* and Carotenoids at High-Light**. Time: 16.52 % variance, p = 0.0002; Strain: 6.80 % variance, p = 0.0581; Interaction: 3.28 % variance, p = 0.7099. d=days.

	Low-Light			High-Light		
	2d	4d	7d	2d	4d	7d
6803 +N vs. B12 +N	ns	ns	ns	ns	ns	ns
6803 +N vs. 6803 -N	*	ns	ns	ns	ns	ns
6803 +N vs. B12 -N	**	ns	ns	ns	ns	ns
B12 +N vs. 6803 -N	***	ns	*	*	ns	ns
B12 +N vs. B12 -N	***	ns	**	ns	ns	ns
6803 -N vs. B12 -N	ns	ns	ns	ns	ns	ns
